# The Immunomodulatory Effect of You-Gui-Wan on *Dermatogoides-pteronyssinus*-Induced Asthma

**DOI:** 10.1155/2012/476060

**Published:** 2012-05-20

**Authors:** Li-Jen Lin, Chin-Che Lin, Shulhn-Der Wang, Yun-Peng Chao, Shung-Te Kao

**Affiliations:** ^1^School of Chinese Medicine, College of Chinese Medicine, China Medical University, Taichung 40402, Taiwan; ^2^Graduate Institute of Chinese Medicine, China Medical University, Taichung 40402, Taiwan; ^3^School of Post-Baccalaureate Chinese Medicine, College of Chinese Medicine, China Medical University, Taichung 40402, Taiwan; ^4^Department of Chemical Engineering, Feng Chia University, Taichung 40724, Taiwan; ^5^Department of Chinese Medicine, China Medical University Hospital, Taichung 40402, Taiwan

## Abstract

The traditional Chinese medicine You-Gui-Wan (YGW) contains ten species of medicinal plants and has been used to improve health in remissive states of asthma for hundreds of years in Asia. However, little is known about the immunomodulatory mechanisms in vivo. Therefore, this study investigated the pathologic and immunologic responses to YGW in mice that had been repeatedly exposed to *Dermatogoides-pteronyssinus* (Der p). YGW reduced Der-p-induced airway hyperresponsiveness and total IgE in serum. It also inhibited eosinophil infiltration by downregulating the protein expression of IL-5 in serum and changed the Th2-bios in BALF by upregulating IL-12. Results of the collagen assay and histopathologic examination showed that YGW reduced airway remodeling in the lung. In addition, after YGW treatment there was a relative decrease in mRNA expression of TGF-**β**1, IL-13, eotaxin, RANTES, and MCP-1 in lung in the YGW group. The results of EMSA and immunohistochemistry revealed that YGW inhibited NF-**κ**B expression in epithelial lung cells. YGW exerts its regulative effects in chronic allergic asthmatic mice via its anti-inflammatory activity and by inhibiting the progression of airway remodeling.

## 1. Introduction

Asthma is a public health problem worldwide, but particularly in developed countries [1, 2]. Allergic asthma is defined as an acute-on-chronic inflammatory disease of the airway with characteristic eosinophilic recruitment, airway hyperresponsiveness (AHR), hyperplasia of goblet cells, mucus hypersecretion, deposition of collagen, smooth muscle cell hypertrophy, and subepithelial fibrosis [3, 4] T-cell subsets such as T_H_1, T_H_2, T_H_17, and T_H_9 cells as well as regulatory T cells regulate immune responses to allergens in the allergic lung. Moreover, asthma is considered a T helper 2 (T_H_2)-cell-driven inflammatory disease [5, 6]. Therefore, drugs that can suppress T_H_2 cytokine production would prove useful as allergen immunotherapy agents [7].

Although corticosteroids and *β*-agonists can improve asthma symptoms, they do not cure the disease [8]. These agents also have side effects, particularly in children. Many drugs used in Traditional Chinese Medicine (TCM) have been used for centuries to treat asthma and are still widely used in modern medical practice in Asian countries. Recently, many studies have provided evidence of the therapeutic mechanism of numerous herbal formulas that are useful for treating allergic asthma [9].

YGW, a standard yang-tonic decoction, has been used clinically by practitioners of TCM for more than 400 years. Many clinical observations indicate that YGW is capable of enhancing the body's immune system to defend against pathogens [10]. Moreover, some of the herbs in YGW have been demonstrated to promote blood circulation and to have cardioprotective effects [11]. In addition, YGW has been traditionally used to regulate remissive states of asthma. However, the mechanisms governing the modulatory effects of YGW in asthma are not known. 

A great deal of information on allergic airway inflammation comes from murine model systems using artificial ovalbumin (OVA) combined with adjuvants [5]. However, OVA is not a major allergen in human asthma. Up to 85% of patients with allergic asthma are sensitized to house dust mite (HDMs) allergens, such as *Dermatogoides*-*pteronyssinus *(Der p) [12]. More than ten allergens are found in mites themselves and their fecal pellets [13–18]. These allergens are classified into diverse protein families with various biological functions [19, 20]. For instance, Group 1 allergens are cysteine proteases that cleave intercellular epithelial tight junctions, permitting the transmission of allergens to the submucosal of antigen-presenting cells. Group 2 allergens are interaction with the innate immune system [21]. Repeated dust mite allergen exposure can activate T_H_2 and T_H_1, which together orchestrate subsequent allergic responses resulting in pulmonary inflammation characteristic of chronic allergic inflammation [22].

We developed a mouse model of allergic asthma by repeatedly exposing BALB/c mice to Der p via intratracheal exposure to assess the pattern of airway remodeling and the alteration of inflammatory cells and cytokines in BALF after treatment with YGW. Finally, this study was to determine the immunomodulatory effect of YGW and understand the mechanisms by which YGW modulates T_H_1-T_H_2 responses at the molecular level. Mice that are chronically exposed to Der p exhibit several symptoms that are similar to those found in patients with allergic asthma, including high levels of immunoglobulin E (IgE) in serum, increased AHR, airway inflammation, and remodeling [22].

## 2. Materials and Methods

### 2.1. Mice and Reagents

Animal experiments were carried out in agreement with the principles outlined by the Institutional Animal Care and Use Committee of the China Medical University (number 97-93-N). Specific pathogen-free, male, 6-to 8-wk-old BALB/c mice were purchased from the National Laboratory Animal Center, Republic of China. The mice were housed in a microisolator cage (Laboratory Products, Inc., Maywood, NJ, USA) and were fed sterile food and water ad libitum. Animal care and all experimental treatments followed the guidelines set-up by the National Science Council of the Republic of China. Lyophilized Der p was used in this study (Allergon, Sweden). The crude Der p preparation was extracted with ether and dialysis with deionized water. The extracts were lyophilized and stored at −80°C before use. LPS concentration in the Der p preparation was <0.96 EU/mg of Der p (Limulus amebocyte lysate test; E-Toxate; Sigma-Aldrich).

### 2.2. YGW Preparation

YGW (batch number 98041022) was supplied by Koda Pharmaceutics Ltd (Taoyuan, Taiwan). This preparation was a mixture of ten common Chinese herbal medicines as indicated in Table 1. In brief, these were extracted with 17.5 L and 12.5 L of boiled water for 1 hour, respectively. Poaching liquid was mixed two times. After filtering the liquid, the dregs of the decoction were removed. The filtered liquid was lyophilized and crushed into a thin powder. The yield of dried extract from starting crude material was estimated to be 36.31 (w/w). YGW was dissolved in distilled water and stored at −20°C before administration to mice.

### 2.3. HPLC Analysis of Standard Materials in Test Samples

**Figure 1:**
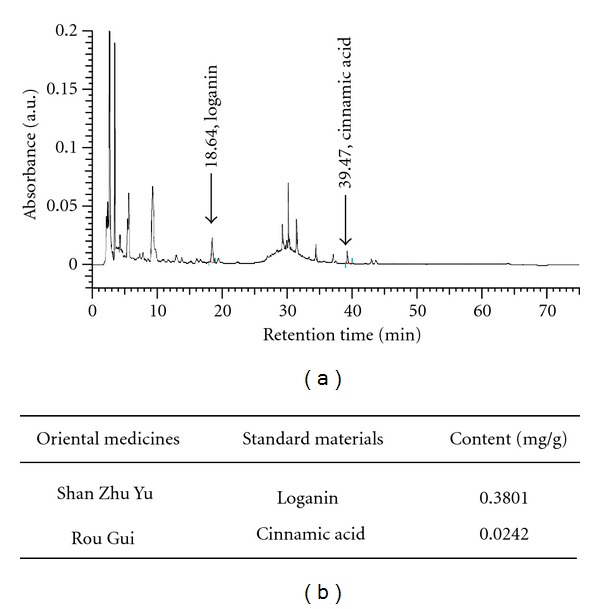
(a) The HPLC chromatogram of YGW. (b) Contents of standard materials in YGW (unit: mg/YGW ex. 1 g).

YGW (1.00 g) was accurately weighed and dissolved in 10 mL of methanol. After sonication for 60 min at room temperature, the solution was filtered through the 0.45 *μ*m membrane filter. YGW was then analyzed by HPLC with Loganin and Cinnamic acid as standards. HPLC analyses were conducted with a system consisting of a Hitachi D-7000 Interface, an L-7100 pump, an L-7420 UV/VIS Detector, an L-7200 Autosampler, and a Mightysil RP-18 (GP) 250 × 4.6 mm (5 *μ*m) column. The mobile phase containing Acetonitrile and 0.03% H_3_PO_4_ (10 : 90) was pumped at a flow rate of 1.0 mL/min and the product (eluant) was detected at 254 nm. A typical HPLC chromatogram is shown in Figure 1(a). The *R* ^2^ values from the calibration curve of two standards were 1.0000 and 0.9999. The quantity of standard materials in YGW was calculated as follows: the amount (mg/g) of standard materials = measured standard materials (mg/*μ*L) × the HPLC injection volume (*μ*L) of YGW × dilution factor/the amount (g) of YGW. The amounts of standard materials in YGW are given in Figure 1(b).

### 2.4. Allergen Challenge and Assessment of Airway Inflammation

 In the Der p group of BALA/c mice (*n* = 6), allergic airway inflammation and remodeling were provoked by subjecting mice to intratracheally administered Der p (1 mg/mL, 50 *μ*L) in phosphate-buffered saline (PBS) once a week for 4 weeks (total 5 doses). In the YGW group, mice were orally administered YGW (1 g/kg) 30 min before exposure to Der p. In parallel experiments, naïve mice were orally administered water and intratracheally administered PBS.

Mice were sacrificed by i.p. injection of xylazine (200 *μ*g/mice) and ketamine (2 mg/mice) three days after the last challenge as reported previously [23]. Serum and BALF samples were collected and stored at −80°C until assay. Differential counts were performed on cytospin preparations (1 × 10^5^ cells/100 *μ*L of BALF) stained with Liu stain (Biotech, Taiwan) in a blind manner after total leukocyte counting.

### 2.5. Measurement of Airway Hyperresponsiveness

 We used Methacholine-induced pause (Penh) values in live mice as a marker of airway responsiveness to bronchoconstrictors. Airway responsiveness was measured in mice using a single-chamber, whole-body plethysmograph (Buxco Electronics, Inc., Troy, NY) according to the manufacture's protocol. The enhanced pause (Penh) variable was used to estimate airway resistance. Mice were serially exposed to increasing doses of nebulized methacholine (0, 3.125, 6.25, 12.5, 25, and 50 mg/mL) (Sigma-Aldrich, St. Louis, MO) in PBS for 3 minutes, respectively, and Penh values were measured for 3 minutes following the end nebulization of methacholine.

### 2.6. Flow Cytometric Analysis

 Monoclonal antibodies used for fluorescence-activated cell-scan (FACScan) staining included PE and/or FITC-conjugated anti-mouse CD4 (BD Pharmingen), FITC-conjugated anti-mouse CD8 (BD Pharmingen), Percp-conjugated anti-mouse CD3 (BD Pharmingen), and FITC-conjugated anti-mouse CD25. BALF cells (1 × 10^5^) were stained with mAb for 30 min on ice. After washing, stained cells were quantified by FACScan (Becton-Dickinson Immunocytometry system, San Jose, CA, USA).

### 2.7. Measurement of Total IgE

Serum samples of total IgE (1 : 2 dilution) were measured by ELISA. Briefly, 96-well plates were coated with purified anti-mouse IgE (2 *μ*g/mL, R35-72; BD Pharmingen). After incubation overnight at 4°C, the plates were washed and then exposed to serum samples in duplicate for 2 h. After washing, biotin anti-mouse IgE Ab (2 *μ*g/mL; BD Pharmingen) was added to individual wells and allowed to incubate for 1 h, followed by washings and the addition of streptavidin-HRP conjugate (1 : 1000 dilution, BD Pharmingen). The plates were washed and developed with a TMB microwell peroxidase substrate system (Kirkegaard & Perry Laboratories, Gaithersburg, MD) and read at OD_450_. Total IgE concentrations were calculated by comparison with commercial mouse IgE standards.

### 2.8. ELISA of Cytokine Levels

 IL-4, IL-5, IL-6, IL-12, and TNF-*α* levels were measured with an ELISA Ready-SET-Go! Kit (eBioscience, San Diego, CA), and IL-13, IFN-*γ*, and TGF-*β* levels were measured with an ELISA DuoSet! Kit (R&D System, Abingdon, UK) according to the manufacturers's protocols. Standards were run in parallel with recombinant cytokines.

### 2.9. Histology and Immunohistochemistry of Lung Specimens

 Paraffin-embedded tissue was cut into 5 *μ*m sections and stained with H&E and periodic acid-Schiff (PAS) stain. Light microscopy was used for histologic assessment. The degree of inflammatory changes was evaluated with a semiquantitative scale of 0–5 for inflammatory cell infiltration, perivascular spaces, and peribronchial spaces. The scale was graded as follows: 0 (none), 1 (minimal, <1%), 2 (slight, 1–25%), 3 (moderate, 26–50%), 4 (moderate/severe, 51–75%), and 5 (severe/high, 76–100%). Meanwhile, change in goblet-cell metaplasia was assessed for mucification and the presence of goblet cells in bronchioles as reported previously [24]. The two scores were summed up for individual lesion in each animal. In the immunohistochemical analysis, the Abs against the RelA subunit of NF-*κ*B (Santa Cruz Biotechnology, Santa Cruz, CA, USA) was used as the primary antibody and HRP-conjugated goat anti-rabbit polycolonal IgG (ZYMED, San Francisco) was used as the secondary antibody. Identification of tissue sites was determined at 100x and 400x magnification.

### 2.10. Collagen Analysis

 Lung tissue (100 mg) from each group was homogenized mechanically in liquid nitrogen and then extracted in 2 mL HBSS. The supernatant collagen was quantified with a Sircol collagen assay kit (Biocolor, Belfast, UK).

### 2.11. Reverse Transcription and Quantitative PCR

Total RNA was extracted from right lung tissue using Trizol reagent following the manufacturer's protocol (Invitrogen Life Technologies). Total RNA was quantified using a spectrophotometer at 260 nm. Total RNA samples (1 *μ*g/mL) were reverse-transcribed with an ImProm-II Reverse Transcription System kit (Promega). Quantitative PCR was carried out with the FastStart Universal SYBR Green Master kit (Roche). *β*-actin was used as a control. The primers (sense and antisense) for each gene were synthesized as follows: *β*-actin, 5′-GGAAATCGTG CGTGACA-3′ and 5′- CACAGGATTCCATACCCAAG-3′; TGF-*β*, 5′-GAGCAACATGTGGAACTCTA C-3′ and 5′-GCAGTGAGCGCTGAAT C-3′; IL-13, 5′-TTAT GGTTGTGTGTTA TTTAAATGAGTCT-3′ and 5′-TGGAGG CTACAGTGAGG T-3′; RANTES, 5′-AGAAGTGGGTTCAAGAATA CAT-3′ and 5′-GGACCGAGTG GGAGTAG-3′; Eotaxin, 5′-ACATGTTACATTTA AGAAATTG GAGTT-3′ and 5′-AGGTCAGCCT GGTCTAC-3′; and MCP-1, 5′-AGAAGGAATGGGTCCAGACATA-3′ and 5′-TTAAG GCATCACAGTCCGAG-3′.

### 2.12. Nuclear Extracts and EMSA

 Nuclear extracts were prepared from left lung tissue according to the manufacturer's instructions (Panomics, Redwood). A LightShift Chemiluminescent EMSA Kit (PIERCE, Rockford) was used to detect NF-*κ*B expression according to the manufacturer's instructions.

### 2.13. Statistical Analysis

Data are presented as means ± SD. Differences between mean values were estimated using a Student's *t*-test. A *P* value <0.05 was considered significant. For comparisons of data that were not normally distributed, a Mann-Whitney *U*-test was performed.

## 3. Results

### 3.1. Effects of YGW on Antiairway Inflammation, Antiairway Hyperresponsiveness, and Downregulation of Total IgE in a Chronic Asthmatic Mouse Model

We used a repetitive Der p challenge experiment to test the effects of YGW on allergen-induced chronic airway inflammation in a mouse model.  Table 2 shows the total and different cell counts in BALF from mice that had been treated with or without YGW during repeated Der p intratracheal inoculation periods (at 1-week intervals). In naïve mice, the cells in BALF were mostly macrophages, and no eosinophils were detected. However, in the Der p group, the numbers of total cells, macrophages, and eosinophils in BALF were markedly higher than those in naïve mice. Numbers of total cells, macrophages and eosinophils in BALF after allergen challenge were significantly lower in mice in the YGW group than these in untreated challenged mice.

**Figure 2:**
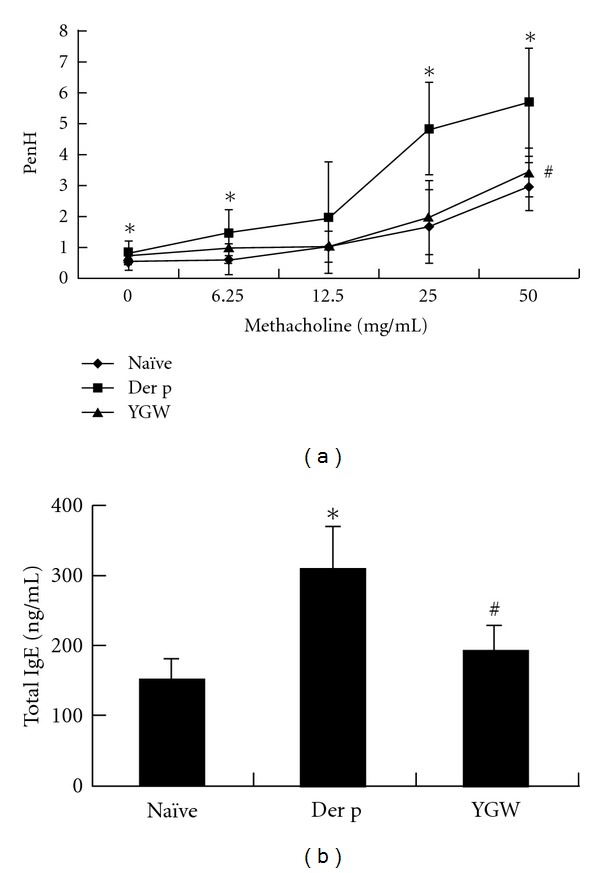
The effects of YGW on antiairway hyperresponsiveness and downregulation of total IgE in a chronic asthmatic mouse model. (a) Airway hyperresponsiveness was determined two days after the last challenge. (b) YGW significantly inhibited the overexpression of IgE in serum. Values represent the mean ± SD of 6 mice. *P < 0.05 (versus naïve group); ^#^ *P* < 0.05 (between nontreated and YGW-treated groups).

We determined the correlation between airway inflammation and airway AHR. Mice in the Der p group had higher Penh values than those in the naïve group. In the YGW group, there was a marked decrease in Penh values at maximal dose of methacholine (50 mg/mL) relative to the Der p group (Figure 2(a)).

To determine the effects of YGW treatment on humoral immune responses, we measured serum total IgE. A significant increase in the levels of serum total IgE was observed in the Der p group relative to the naïve group (Figure 2(b)). After treatments with YGW in the sensitization period, the serum concentrations of total IgE decreased significantly.

### 3.2. YGW Attenuates Der-p-Induced Lung Pathology

**Figure 3:**
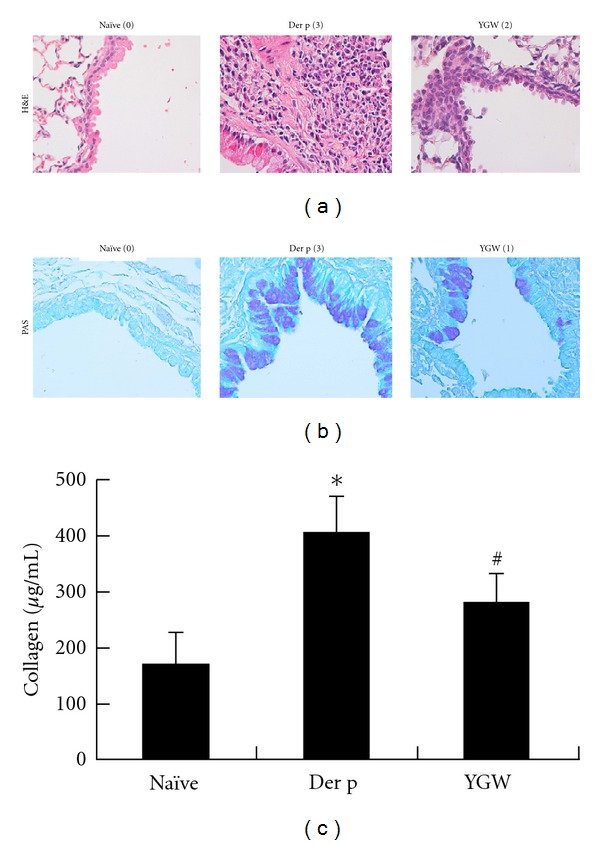
The effects of YGW on Der-p-induced airway inflammation, goblet cell hyperplasia, mucus hypersecretion, and collagen deposition in lung tissue of mice. Airway hyperresponsiveness was determined three days after the last challenge. Histopathologic studies of inflammatory cells around the blood vessels (H&E stain; original magnification, ×400) (a) and activity of goblet cells (PAS; original magnification, ×400) in airway epithelial cells (b). Degrees of airway inflammation and goblet cell hyperplasia were estimated as described in Section 2. (c) Collagen levels in lung tissue were significantly higher in the Der p group. Values represent the means ± SD of 6 mice. *P < 0.05 (versus naïve group); ^#^ *P* < 0.05 (between nontreated and YGW-treated groups). Values represent the means ± SD of 6 mice.

The characteristic features of asthmatic airway are cell inflammation, the presence of hyperplastic goblet cells, mucus secretion, and collagen deposition. The left lungs of mice were histologically examined 72 h after the final antigen challenge. Histological sections of lung tissue from Der p-challenged mice exhibited increased airway inflammation, matrix deposition in subepithelial regions, and excessive mucus secretion from hyperplastic goblet cells (Figure 3), relative to mice in the naïve group. In contrast, mice that had been treated with YGW showed significantly less airway inflammation, fewer PAS-positive cells, and less collagen deposition than mice in the Der p group.

### 3.3. The Effect of YGW on T-Cell Subsets in BALF of Challenged Mice

**Figure 4:**
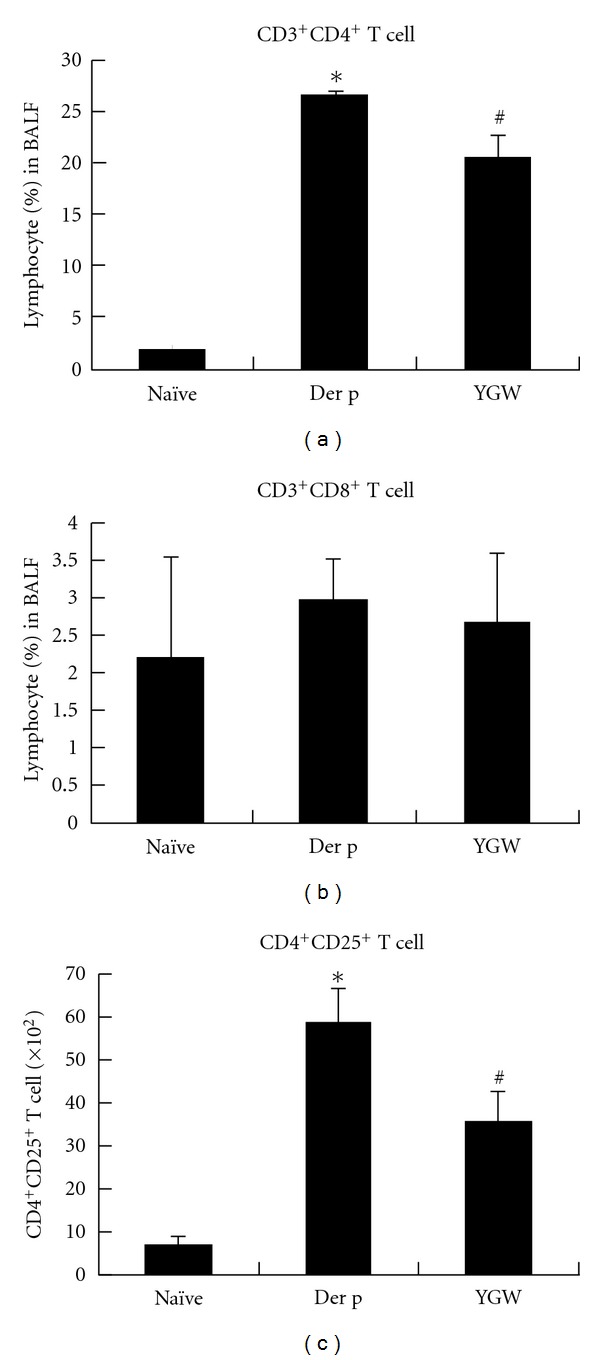
The effect of YGW on T-cell subsets in BALF of challenged mice. (a) CD3^+^/CD4^+^, (b) CD3^+^/CD8^+^, and (c) CD4^+^/CD25^+^ lymphocyte levels were determined by flow cytometry with immunofluorescence of monoclonal antibodies. Data are representative of the results from three separate experiments. *P < 0.05 (versus naïve group); ^#^ *P* < 0.05 (between nontreated and YGW-treated groups).

The effect of YGW on the percentage change of T-cell subsets was determined by flow cytometry with immunofluorescence of monoclonal antibodies to CD3^+^, CD4^+^, CD8^+^, and CD25^+^, respectively (Figure 4). There was a significant increase in the percentage of CD3^+^/CD4^+^ and numbers of CD4^+^/CD25^+^ lymphocytes in Der p group mice relative to the naïve group. YGW group mice showed a significantly lower percentage of CD3^+^/CD4^+^ and CD4^+^/CD25^+^ lymphocytes than Der p mice. However, there was no significant difference in CD3^+^/CD8^+^ lymphocyte levels in BALF among the three groups of mice.

### 3.4. YGW Attenuates Cytokine Production in BALF and Serum

**Figure 5:**
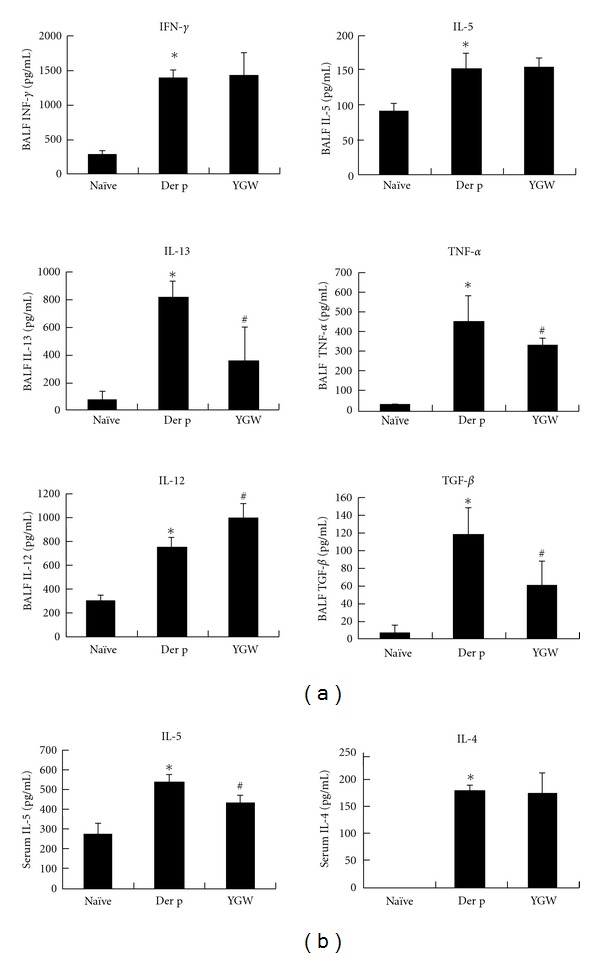
The expression levels of IL-13, TGF-*β*, and TNF-*α* in BALF were reduced by YGW, but levels of IL-12 increased. YGW resulted in reduced production of IL-5 cytokine in serum. *P < 0.05 (versus naïve group); ^#^ *P* < 0.05 (between nontreated and YGW-treated groups).

To determine the possible effect of YGW on T-cell responses, we evaluated the effects of YGW on T-cell cytokine secretion in BALF and serum. The production of IL-4, IL-5, IL-12, IL-13, TNF-*α*, TGF-*β*, and IFN-*γ* was analyzed by ELISA (Figure 5). YGW significantly attenuated the expression of Der-p-induced IL-13, TNF-*α*, and TGF-*β* in BALF but resulted in an increase in IL-12 production relative to mice in the Der p group. YGW group mice also showed significantly lower levels of IL-5 in serum, but not in BALF, than mice in the Der p group. Similarly, there were no changes in IL-4 or INF-*γ* levels among the YGW groups of mice.

### 3.5. Immunoregulation of YGW on Der-p-Induced Proinflammatory Cytokine and Chemokine Gene Expression in Lung Tissues

**Figure 6:**
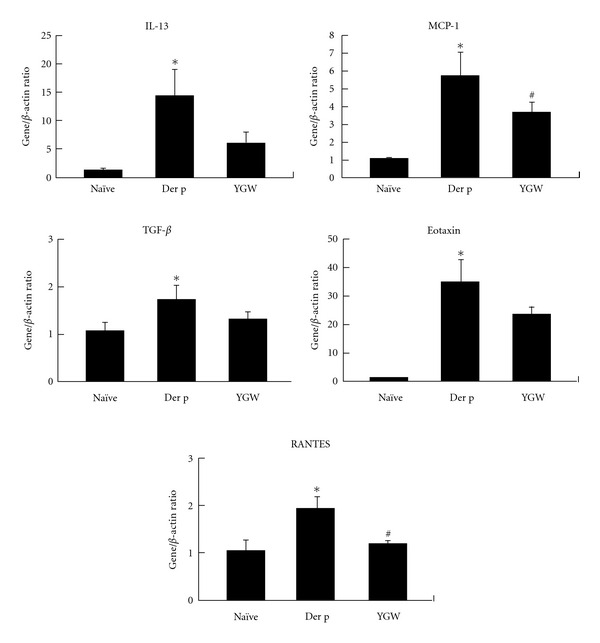
Real-time PCR analysis indicated that YGW exerts its suppressive effects by regulating the expression of T_H_2-related genes and chemokines in lung tissue. This real-time PCR profile was representative of three independent experiments.

 After Der p allergen challenge, the expression levels of IL-13, Eotaxin, RANTES, MCP-1, and TGF-*β* mRNA in lung tissues from the Der p group were higher than those in the naïve group (Figure 6). Lung tissue from mice pretreated with YGW showed significantly lower levels of IL-13, Eotaxin, RANTES, MCP-1, and TGF-*β* synthesis than lung tissues from mice in the Der p group.

### 3.6. NF-*κ*B Activation Was Suppressed in Der-p-Treated Lung Tissues by YGW

**Figure 7:**
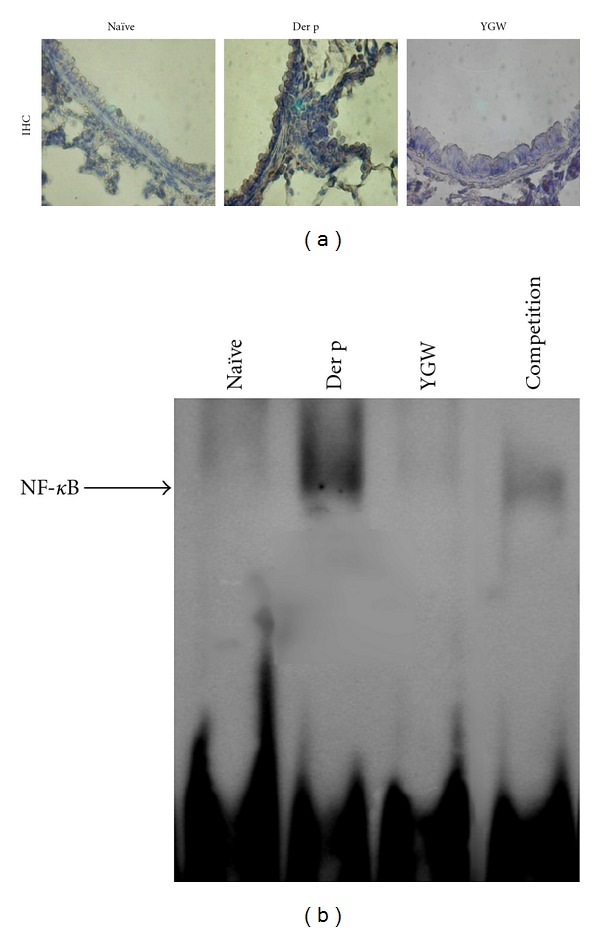
(a) In the immnuohistochemistry assay, YGW inhibited the expression of NF-*κ*B (Rel A; ×400) in bronchiolar epithelial cells of mice after repetitive Der p challenge. (b) EMSA showed that YGW decreased NF-*κ*B activation in whole lung tissue after Der p stimulation. The *arrow* indicates the specific DNA-probe complex. Data are representative of results from three separate experiments.

To test whether the inhibitory effect of YGW on Der-p-challenged animals leads to NF-*κ*B activation, the effects of YGW on expression of NF-*κ*B were examined by immunohistochemistry and its effects on NF-*κ*B-specific DNA-protein binding activity were examined using EMSA. Immunohistochemistry staining showed that YGW inhibited the expression of NF-*κ*B in bronchiolar epithelial cells of Der-p-challenged mice (Figure 7(a)). EMSA revealed that YGW inhibited NF-*κ*B-specific DNA-protein binding activity in lung tissues (Figure 7(b)).

## 4. Discussion

In this study, we provide evidence for the immunoregulatory effects of YGW on allergen-induced airway inflammation and airway hypersensitivity in an established murine model of chronic allergic asthma.

We demonstrated that oral administration of YGW suppressed AHR and reduced the degree of airway inflammation and remodeling. YGW possesses anti-inflammatory effects as evidenced by the reduction in total numbers of cells and the percent of macrophages and eosinophils in BALF relative to those seen in Der p group mice. Moreover, YGW also reduced inflammation and the number of hyperplastic goblet cells as evidenced by lung histology analysis as well as attenuated deposition of collagen. Therefore, YGW appears to suppress AHR by protecting against airway inflammation, eosinophilia infiltration, and airway remodeling.

It is well established that elevated levels of serum IgE correlate with the incidence or severity of asthma. Therefore, interfering with synthesis of IgE or inhibiting its action has recently become a novel therapeutic approach for development of immunological drugs in asthma, such as omalizumab (Xolair) [3]. In the present study, YGW treatment significantly decreased total IgE in serum. Furthermore, we also found that YGW treatment resulted in a reduction in IgG1 levels and in slight elevation of IgG2a/2b levels (data not show).

T cells with a T_H_2-like phenotype are thought to play an important role in orchestrating the asthmatic inflammatory response [25]. Therefore, the immunomodulatory role of YGW in allergen-sensitized mice may lie in its ability to regulate T-cell activation. In this study, flow cytometry analysis showed that YGW led to a decrease in the percentage of the CD3^+^CD4^+^ T-cell subset in BALF, but not the CD3^+^CD8^+^ T-cell subset. On the other hand, CD25^+^CD4^+^ regulatory T cells suppress immune responses and are believed to play roles in preventing autoimmune diseases [26]. It has been reported that regulatory T cells downregulate CD25^−^CD4^+^ T-cell-mediated production of IL-12 in antigen-presenting cells [27]. In our study, we found that YGW reduced the number of CD4^+^CD25^+^ T cells in BALF, suggesting that YGW may block this feedback system and shift Th2-bios by increasing IL-12 levels. Further studies are needed to verify the effect of YGW on changing T-cell subsets during allergic inflammation.

Blood and tissue eosinophilia are hallmarks of allergic rhinitis, atopic dermatitis, and atopic asthma. Eosinophils are recruited from the circulation to inflammatory tissues in response to allergic stimuli [28]. Moreover, eosinophils contribute to the symptomatology of asthma by releasing granule-stored cationic proteins and proinflammatory mediators, including cytokines and chemokines [29]. Therefore, the eosinophil is thought to be a key effector cell in the pathogenesis of allergic disease [29]. Notably, the important regulators of eosinophil trafficking are T_H_2 cytokines such as IL-4, IL-5, and IL-13 [29, 30], although chemokines, eotaxis, and RANTES are also involved in recruitment of eosinophils [31]. In addition, only IL-5 is specific for terminal differentiation, growth, and survival of eosinophils [32, 33]. Thus, suppression of these cytokines and chemokines may be effective in reducing eosinophil infiltration into lung, thereby alleviating allergic asthmatic inflammation. In addition, RANTES and MCP-1 are also involved in recruitment of monocytes [34]. In our present student we showed that YGW treatment decreased the secretion of IL-5 in serum and IL-13 in lung tissue but did not affect IL-4 levels in serum. Meanwhile, YGW treatment downregulated mRNA expression of IL-13, eotaxin, RANTES, and MCP-1 in lung tissue. Therefore, we speculate that YGW treatment reduces eosinophil infiltration into lung by modulating T_H_2 responses at the molecular level.

In asthmatics, one important feature of airway remodeling is subepithelial fibrosis [35]. TGF-*β* is a profibrogenic growth factor, which stimulates the differentiation of fibroblast precursors to myofibroblast cells, which in turn induces their proliferation [36]. Moreover, it has been shown to elicit the expression of MMPs and TIMPs [37]. Meanwhile, many studies have shown that TGF-*β* and eosinophils play important roles in the development of airway remodeling [38–41].

The rate of apoptosis of bronchial epithelial cells is significantly higher in asthma patients than that in healthy people, and TNF-*α* is suggested to play a crucial role in this process [42, 43]. In addition, TNF-*α* can regulate overproduction of ECM proteins by inducing epithelial cells, fibroblasts, and ASM cells [44]. Simultaneously, TNF-*α* and IL-1*β* can induce the synthesis of eotaxin [45] and RANTES [46] in human lung epithelial cells.

 We found that YGW treatment resulted in decreased secretion of TGF-*β* and TNF-*α* in BALF and the downregulation of mRNA expression of TGF-*β* in lung tissues. Therefore, we propose that YGW treatment reduces airway subepithelial fibrosis by downregulating the expression of TGF-*β* and TNF-*α*.

NF-*κ*B is capable of regulating T_H_2 cell differentiation and producing proinflammatory mediators that are required for induction of allergic airway inflammation [47]. Bureau et al. [48] reported that bronchial epithelial and/or airway immune cells displayed augmented NF-*κ*B activity in OVA/OVA mice [48]. Therefore, targeting of the NF-*κ*B signaling pathway may be a promising therapy for asthma [48]. However, whether the immunoregulatory activity of YGW against asthma is connected with a diminution of NF-*κ*B activity in the lung is not clear. To address these issues, we used immunohistochemistry and EMSA to detect NF-*κ*B expression and activation in lung of repeatedly Der p challenge mice. We found that oral administration of YGW markedly reduced NF-*κ*B expression, predominantly in bronchial epithelium. EMSA analysis also revealed a decrease in NF-*κ*B activation in the whole lung (Figure 7). Taken together, we speculate that inhibition of NF-*κ*B activation directly or indirectly attenuated IL-5, IL-13, eotaxin, RANTES, TNF-*α*, and TGF-*β* gene expression, eosinophilia infiltration, mucus production, collagen deposition, and allergic lung inflammation.

In conclusion, YGW-induced attenuation of AHR, remodeling, and inflammation in this model of chronic asthma is associated with specific downregulation of T_H_2 cytokines and inhibition of NF-*κ*B activation within the bronchial epithelium in lung. To the best of our knowledge, this is the first study to show that YGW has immunoregulation effects in remissive states of asthma. The pharmacologically active components of YGW, however, have not yet been characterized. Further research will be required to dissect the mechanisms of action of this formula.

## Figures and Tables

**Table 1:** Components of herbal medicines in YGW.

TCM materia medica	Pharmacognostic nomenclature	Amount (g)	Part used
(1) Shou Di Huang	*Rehmanniae glutinosa*	8.0	root
(2) Shan Yao	*Dioscorea opposita*	4.0	root
(3) Shan Zhu Yu	*Cornus officinalis*	4.0	fruit
(4) Gou Qi Zi	*Lycium chinensis*	4.0	fruit
(5) Yu Si Zi	*Cuscuta chinensis*	4.0	seed
(6) Lu Jiao Jiao	*Cervus elaphus Linnaeus*	3.0	horn
(7) Du Zhong	*Eucommia ulmoides*	4.0	cortex
(8) Dang Gui	*Angelica sinensis*	3.0	root
(9) Rou Gui	*Cinnamomun cassia*	2.0	cortex
(10) Fu Zi	*Aconitum Carmichaeli*	2.0	root

Total amounts		38.0	

**Table 2:** The total cell number and cellular distributions in BALF of mice 72 h after repetitive Der p challenge.

	Total cells (×10^4^/mL)	Macrophages (%)	Lymphocytes (%)	Neutrophiles (%)	Eosinophiles (%)
Naïve	13.6 ± 2.19	12.60 ± 1.91 (92.8 ± 3.27%)	0.52 ± 0.21 (4.2 ± 1.79%)	0.48 ± 0.37 (3.4 ± 2.51%)	0 (0%)
Der p	69.0 ± 12.25*	52.99 ± 9.25* (77.0 ± 4.69%)	4.49 ± 1.26* (6.6 ± 1.81%)	6.81 ± 5.05* (9.4 ± 6.43%)	4.68 ± 1.50* (7.0 ± 2.55%)
YGW	39.2 ± 3.11^#^	33.17 ± 4.00^#^ (84.6 ± 7.23%)	2.99 ± 1.53 (7.6 ± 3.78%)	2.59 ± 1.20 (6.6 ± 3.21%)	0.52 ± 0.56^#^ (1.4 ± 1.52%)

## Abbreviations

TCM: Traditional Chinese medicine
YGW: You-Gui-Wan
HDM: house dust mite
Der p: Dermatophagoides pteronyssinus
i.t.: intratracheal
BALF: Bronchoalveolar lavage fluid (s).
